# Clinical Applications of End-to-Side Neurorrhaphy: An Update

**DOI:** 10.1155/2014/646128

**Published:** 2014-07-20

**Authors:** Pierluigi Tos, Giulia Colzani, Davide Ciclamini, Paolo Titolo, Pierfrancesco Pugliese, Stefano Artiaco

**Affiliations:** Department of Orthopaedics, Traumatology and Rehabilitation, Microsurgery Unit, AO Città della Salute e della Scienza, Orthopaedic and Trauma Center, Via Zuretti 29, 10126 Turin, Italy

## Abstract

End-to-side neurorrhaphy constitutes an interesting option to regain nerve function after damage in selected cases, in which conventional techniques are not feasible. In the last twenty years, many experimental and clinical studies have been conducted in order to understand the biological mechanisms and to test the effectiveness of this technique, with contrasting results. In this updated review, we consider the state of the art about end-to-side coaptation, focusing on all the current clinical applications, such as sensory and mixed nerve repair, treatment of facial palsy, and brachial plexus injuries and painful neuromas management.

## 1. Introduction

It is currently accepted that autologous nerve grafting is the gold standard for nerve repair in presence of major loss of substance after injury. Sometimes however this technique is not feasible for many reasons, like the limited amount of obtainable graft tissue in case of large and extended loss of substance, the unavailability of the proximal nerve stump, and the morbidity at the donor site. Many surgical alternatives have been proposed over time, including the use of synthetic and biological tubulization, the application of cultured Schwann cells, and the end-to-side neurorrhaphy [1].

End-to-side neurorrhaphy was first described by Létiévant in the “Traité des Sections Nerveuses” in 1873 [2]. In the following years this technique inspired several other clinical and experimental studies but then was quickly abandoned due to poor clinical results probably related to the use of nonmicrosurgical instruments and techniques, until a new interest appeared at the beginning of the 1990s with the publication of the studies of Lykissas et al. [1, 3]. Thereafter a series of researches have been conducted, in order to further understand the mechanisms at the base of this way of nerve regeneration and to improve clinical applications and results.

The possibility to regain nerve function after damage even if the proximal stump is not available is the aim of this technique, based on the concept that collateral axonal sprouting from a healthy neighbor donor nerve can involve a distal stump of a transected nerve, if they are sutured in end-to-side fashion [3].

The phenomenon of the sprouting from the terminal portion of the proximal stump of a damaged nerve is a well-known concept, induced by molecular changes in the microenvironment in which the nerve lesion occurred, sustained by Wallerian degeneration, interruption of the normal neuronal turnover and inflammatory local response. The possibility that a healthy adult axon could generate collateral sprouting to reinnervate the target organ of another nerve was considered unlikely for a long time. In the last twenty years several studies were conducted on this field and finally demonstrated the existence of this phenomenon following end-to-side neurorrhaphy. One of the most unsolved questions is the origin of the regenerating axons after end-to-side neurorrhaphy. Some researchers support the idea that they could come from the donor nerve's axons which have been injured during surgery by opening an epineural window, especially in case of motor ones [4, 5].

Consequently, another point of discussion is whether the technique of end-to-side suture may influence the amount of regenerating axons originating from the donor nerve. Regenerating axons may penetrate the connective layers of the nerve (epineurium, perineurium, and endoneurium) [6]. Then, in clinical practice, most authors perform an epineural window in the donor nerve before suture creating a small not standardized nerve damage which enhances the opportunity of axonal collateral sprouting. In experimental study on rats, Viterbo et al. demonstrated that there is no difference in the amount of regenerating axons after end-to-side nerve suture with and without epineural window [7], and Lundborg et al. confirmed these experimental results [8]. Subsequently, Viterbo et al. investigated nerve regeneration after end-to-side nerve suture with and without perineural window reporting similar results [9]. Other experimental studies suggested that the ingrowth of regenerating axons is significantly enhanced by breaching the epiperineural sheath at the coaptation site [10] and that an epineural window followed by terminolateral suture without damage of underlying perineurium improves functional outcomes of end-to-side nerve suture [1]. In fact, this procedure stimulates nerve regeneration with better results without creating a significant damage in the donor nerve [11–13].

The aim of this study is to present the clinical applications of end-to-side neurorrhaphy on the basis of current knowledge and the literature review.

## 2. Clinical Applications

From the analysis of the literature of the last two decades we found an increasing interest in the application of this technique in a wide pattern of peripheral nerve damages, even if in the form of case reports and small clinical series. No randomized clinical trials have been performed until now, in order to compare end-to-side coaptation to other reconstructive techniques. Despite the encouraging results that come from experimental studies, the small series found in the literature are difficult to compare and the contrasting clinical outcomes do not permit to assume yet the real effectiveness of this technique. The analysis of the most documented clinical applications includes repair of digital sensory nerves lesions treatment of brachial plexus and facial nerve injuries, mixed nerves lesions, and painful neuromas.

### 2.1. Sensory Nerves

#### 2.1.1. Digital Nerves

A small number of retrospective clinical studies about the application of end-to-side neurorrhaphy for treatment of sensory nerve lesions can be found in the literatures [14–19].

In his comprehensive study on the use of end-to-side nerve suture in clinical practice, Mennen described five cases of palmar digital nerves terminolateral neurorrhaphy reporting four good results based on the Highet British Medical Research Council test [16]. Other studies confirmed these favorable results.

According to the Highet British Medical Research Council scale, Pelissier reported positive results in five out of six patients with S3+ sensory recovery and a mean 2pd of 10 mm (9–12 mm) [17]. Voche and Quattara reported the same degree of recovery S3+ in nine patients of his clinical series with a mean 2pd of 8 mm (range 6–11 mm) [18].

In our personal experience we reported sensory recovery in seven patients with end-to-side nerve suture of collateral digital nerves observing S3+ recovery in six cases and S3 in one case with a mean 2pd of 12.5 mm (range 8–18 mm) [19]. Moreover we evaluated the Semmes-Weinstein monofilament test observing a 3.61 sensory threshold in two cases and a 4.61 threshold in five cases.

In line with these findings, end-to-side technique can reasonably be considered as a valid treatment of nerve repair in selected cases as an alternative to biologic or synthetic tubulization and autograft, when direct suture is impossible for the length of the gap, in traumatic and post surgical conditions.

#### 2.1.2. Other Sensory Nerves

In 1993 Viterbo et al. reported restoration of sensitivity in the region on sural nerve, harvested as a graft, after end-to-side coaptation of its distal stump to the superficial peroneal nerve in two patients [20]. A study of Santamaria compared end-to-side and end-to-end neurorrhaphy in cases of innervated radial forearm flaps for hemiglossectomy reconstruction, revealing a better sensory recovery using end-to-end procedure [21]. In order to ameliorate sensibility in paraplegics, to avoid the formation of pressure ulcers, Viterbo and Ripari used sural nerve graft linking the intercostal nerves above the level of cord injury and the sciatic nerve below using end-to-side neurorrhaphy in two patients [22].

### 2.2. Brachial Plexus

The first case of end-to-side neurorrhaphy applied on brachial plexus injury was reported by Pienaar et al. in 1995, which sutured C5 and C6 roots to the phrenic nerve [23]. Heterogeneous results can be found in the literature, with the application of this technique in a wide range of conditions [16, 24–28].

In 2003, Mennen published a series of eight cases of brachial plexus repair in end-to-side but reported neither the level of the lesion nor the associated procedures, showing moderate motor and sensory recovery according to the Medical Research Council evaluation scale [16]. Amr and Moharram described eleven cases of repair of roots ruptures with suture to phrenic and spinal accessory nerves, contralateral C7, and other interplexus roots or cords. Functional recovery was satisfying in all but one case in which deterioration of the donor muscle was observed, which improved one year later [24]. The study of Pienaar et al. on nine patients with eight traumatic and one obstetric incomplete brachial plexus lesions showed a partial sensory recovery only in two cases and no useful motor recovery [27]. Haninec et al. reported a homogeneous series of incomplete brachial plexus injuries treated by intraplexual donor nerves (radial, median, and ulnar) on the axillary nerve and musculocutaneous nerve. The overall success rate of motor recovery after end-to-side nerve suture was 43.5%. Reinnervation of deltoid muscle was observed in 47.6% of patients and no recovery was seen as for musculocutaneous nerve repair, with a motor recovery on the deltoid muscle in 64% of the cases [26]. Battiston et al. reported eleven patients that underwent various end-to-side nerve repairs, almost always in order to support shoulder function in abduction and external rotation movements. This study did not demonstrate significant benefits in primary reconstructive surgery for traumatic injuries in adults. In one of these patients an end-to-side coaptation of the hypoglossal to suprascapularis nerve resulted in the recovery of some active shoulder abduction, in contrast to previous study of Ferraresi et al. [25, 28, 29]. The usefulness of this technique applied on brachial plexus injuries still remains questionable, overall in adults, where standard neurotizations are more reliable. However, this technique can be occasionally used to support conventional procedures when other options are not feasible.

### 2.3. Mixed Nerves

**Figure 1:**
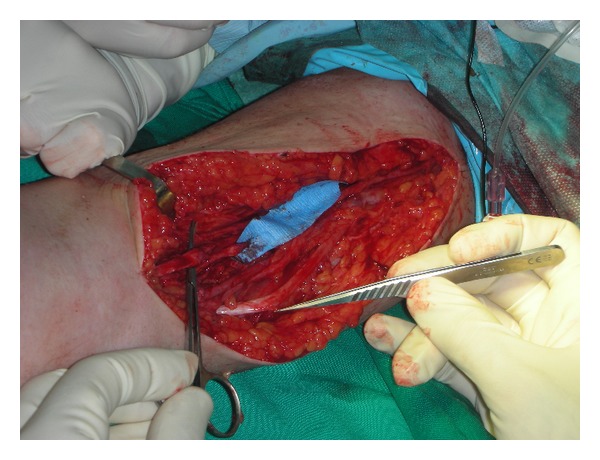
Median nerve section with extensive loss of substance at the forearm.

**Figure 2:**
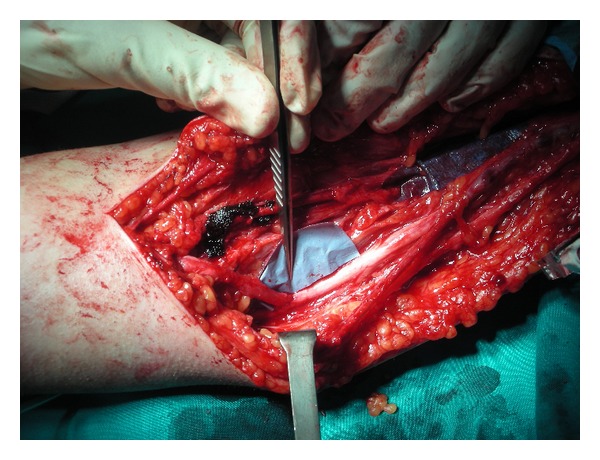
End-to-side neurorrhaphy: median to ulnar nerve.

Treatment of mixed nerves defects can be easily performed by end-to-side coaptation, particularly between median and ulnar nerves at the forearm (Figures 1 and 2). Mennen reported thirty-three cases of ulnar to median nerve suture and seven of median to ulnar nerve suture, with satisfying sensory recovery despite poor motor recovery [16]. This conformed previous findings by Luo et al. [30]. Improvement in protective sensibility was also demonstrated by some authors performing median to ulnar end-to-side coaptation in several cases reported in the literature with long nerve defects [31–34]. In such cases only partial motor recovery was occasionally seen. Probably the cause has to be found in the difficulty of regenerating axons to match properly sensory and motor fibers in mixed nerve [31, 33]. In these situations a neurotization seems to be a more reliable surgical option.

### 2.4. Facial Nerve

The first application of end-to-side coaptation in facial palsy was reported by Viterbo in 1993, who performed a cross-facial nerve graft transplantation, obtaining reinnervation in selected patients. Sural grafts were used to connect both buccal branches and both temporozygomatic branches with end-to-side sutures in both endings [35]. Subsequent papers published by Scaramella and Smith confirmed the possibility to use this kind of grafts to cross the face with minimal residual deficits on the donor site [36, 37].

At present the hypoglossal-facial anastomosis is a reliable procedure for the reanimation of a long-lasting peripheral facial nerve paralysis [38]. The use of an interposition graft and its end-to-side anastomosis to the hypoglossal nerve allows the preservation of the tongue function requires two anastomosis sites and a free second donor nerve. A rat model investigated such configuration of end-to-side nerve suture and showed that axonal regeneration through interpositional was bidirectional and preferentially directed towards the injured side clarifying the basis of clinical application [39].

Favorable results on the treatment of facial paralysis were also reported by Frey et al. with combination of end-to-end and end-to-side neurorrhaphies in three out of seven patients [40]. In our opinion these results could be explained by the fact that the facial nerve is a pure motor structure, so regenerating axons after end-to-side suture can directly and exclusively reach the motor target.

### 2.5. Painful Neuromas

The concept of end-to-side nerve suture has also been applied to the prevention and treatment of painful neuromas secondary to damage of sensory nerves. This application is based on the possibility to give a new pathway and target organ to regenerating axons of the injured nerve. Al-Qattan reported his experience on the use of end-to-side coaptation for prevention and treatment of painful neuromas of superficial branches of the radial nerve: the two ends of the injured sensory nerve branch were sutured in end-to-side fashion on a contiguous branch of the superficial radial nerve. The author obtained positive results in eight patients with preliminary application of such technique [41]. Favorable results were also confirmed by Aszmann et al. in sixteen out of seventeen patients affected by painful neuromas of sensory nerves located at the upper and lower limbs. No motor sensory deficit was seen in the recipient nerves in this case series [42]. An alternative technique for neuroma management has been experimented on a rat model by Isaacs et al. and Adelson et al. consisting in end-to-side “jump grafts” which bypass a neuroma incontinuity. In such technique the proximal repair is a conventional end-to-side suture while in the distal connection the axons in the graft must enter the side of the distal nerve in a reverse end-to-side neurotization process [43, 44]. The concept of reverse end-to-side nerve suture with axonal supercharging of the recipient nerve has been also experimentally investigated in rat model with complete and incomplete sciatic nerve injuries. These studies demonstrated that functional recovery after peripheral nerve injury was promoted by axonal augmentation [45–47]. Nevertheless, to our knowledge, no clinical applications of such technique have been reported up to now.

## 3. Discussion

The research conducted in the last twenty years demonstrated that end-to-side coaptation may be an effective mean of nerve reconstruction in case of loss of substance superior to 3-4 cm, or when the proximal nerve stump or nerve donors are not available. Nevertheless, a discrepancy between experimental and clinical results still exists, confirming the great complexity of the mechanisms of nerve regeneration that are far to be completely understood and the difficulty to transfer the results obtained in laboratory to the clinical application [48]. It has also been demonstrated that axon growth after end-to-side neurorrhaphy is slower compared to end-to-end suture, and this can be one of the possible reasons for unsatisfactory outcomes [49, 50]. Experimental studies have shown that physical and chemical agents could stimulate nerve regeneration after end-to-side coaptation, as phototherapy [51], FK506 [52], and acetyl-L-carnitine [53]. These findings can address future research in order to improve the effectiveness of this technique. However, it is largely agreed that at present end-to-side neurorrhaphy could not substitute standard techniques in most cases, as brachial plexus repair, but can be considered a valid therapeutic option in selected situations, optionally in combination with other strategies, in case of failure of other previous attempts of nerve repair or whenever other approaches are not feasible.
